# Spectrum of genetic variants in bilateral sensorineural hearing loss

**DOI:** 10.3389/fgene.2024.1314535

**Published:** 2024-02-12

**Authors:** Amanat Ali, Mohammed Tabouni, Praseetha Kizhakkedath, Ibrahim Baydoun, Mushal Allam, Anne John, Faiza Busafared, Ayesha Alnuaimi, Fatma Al-Jasmi, Hiba Alblooshi

**Affiliations:** ^1^ Department of Genetics and Genomics, College of Medicine and Health Sciences, United Arab Emirates University, Al Ain, United Arab Emirates; ^2^ Department of Otolaryngology, Al Kuwait Hospital, Dubai, United Arab Emirates; ^3^ Department of Pediatrics, Tawam Hospital, Al Ain, United Arab Emirates

**Keywords:** bilateral sensorineural hearing loss, genetic variants, MYO15A gene, SLC26A4 gene, GJB2 gene, CDH23 gene, whole-exome sequencing, genomics

## Abstract

**Background:** Hearing loss (HL) is an impairment of auditory function with identified genetic forms that can be syndromic (30%) or non-syndromic (70%). HL is genetically heterogeneous, with more than 1,000 variants across 150 causative genes identified to date. The genetic diagnostic rate varies significantly depending on the population being tested. Countries with a considerably high rate of consanguinity provide a unique resource for studying rare forms of recessive HL. In this study, we identified genetic variants associated with bilateral sensorineural HL (SNHL) using whole-exome sequencing (WES) in 11 families residing in the United Arab Emirates (UAE).

**Results**: We established the molecular diagnosis in six probands, with six different pathogenic or likely pathogenic variants in the genes *MYO15A*, *SLC26A4*, and *GJB2*. One novel nonsense variant, *MYO15A*:*p*.Tyr1962Ter*, was identified in a homozygous state in one family, which has not been reported in any public database. *SLC26A4* and *GJB2* were found to be the most frequently associated genes in this study. In addition, six variants of uncertain significance (VUS) were detected in five probands in the genes *CDH23*, *COL11A1*, *ADGRV1*, *NLRP3*, and *GDF6*. In total, 12 variants were observed in eight genes. Among these variants, eight missense variants (66.7%), three nonsense variants (25.0%), and one frameshift (8.3%) were identified. The overall diagnostic rate of this study was 54.5%. Approximately 45.5% of the patients in this study came from consanguineous families.

**Conclusion:** Understanding the genetic basis of HL provides insight for the clinical diagnosis of hearing impairment cases through the utilization of next-generation sequencing (NGS). Our findings contribute to the knowledge of the heterogeneous genetic profile of HL, especially in a population with a high rate of consanguineous marriage in the Arab population.

## Introduction

Hearing loss (HL) is the most prevalent sensory disorder worldwide. It affects both children and adults in either partial or complete form of hearing impairment. It is estimated that 2 in 1,000 children are born with HL, and two-thirds of individuals over the age of 70 years have a hearing impairment (Thompson et al., 2001; Lin et al., 2011). HL onset and progression are highly diverse. It can be congenital or late-onset, temporary or permanent, affecting either one (unilateral) or both ears (bilateral) at various degrees (Müller and Barr-Gillespie, 2015). Approximately 70% of all hereditary HL is non-syndromic, while 30% is syndromic (Yan et al., 2016). It is estimated that 80% of the genetic cases of non-syndromic HL are inherited in an autosomal recessive manner and are typically congenital or prelingual (Lammens et al., 2013). Autosomal dominant forms of non-syndromic HL, on the other hand, typically have a later onset and tend to be progressive and account for the remaining 20% of the genetic cases. X-linked and mitochondrial inheritance cases are rare and may account together for up to 2% of the genetic cases of non-syndromic HL (Smith et al., 2005; Angeli et al., 2012).

Genetic testing has become an essential component of the diagnostic process, especially for children with HL, in the last decade. HL is genetically heterogeneous, and currently, over 150 causative genes have been identified. More than 1,000 variants in genes, including *SLC26A4* (OMIM 605646), *CDH23* (OMIM 601067), *GJB2* (OMIM 220290), *STRC* (OMIM 606440), and *OTOF* (OMIM 603681), have been found to be associated with non-syndromic HL (Chari and Chan, 2017). The vast majority of these have been reported primarily in consanguineous families. Many genes and variants have distinctive clinical phenotypes and are often generally categorized into those mainly linked with non-syndromic progressive sensorineural HL (SNHL), non-syndromic stable SNHL, and syndromic SNHL. Therefore, understanding the genes responsible for a child’s HL can be helpful for predicting how this condition will progress.

In general, genetic factors have a central role in the etiology and pathophysiology of HL, and thus, genetic testing for HL has a significant clinical value for its diagnosis and management. With the recent and continued advancement in next-generation sequencing (NGS), comprehensive genetic testing for HL is now more feasible and cost-effective for clinical practice and research (Lin et al., 2012; Shearer and Smith, 2012; Korver et al., 2017). Therefore, more genetic variants and loci are likely to be associated with HL in the near future, and more ambiguous events in the disease pathophysiology could hopefully be resolved.

In this study, we recruited 11 families who had at least one affected individual with bilateral SNHL with an aim to find the genetic causes of bilateral SNHL in affected patients. This study also helped in establishing the contribution of multiple HL genes in the etiology of bilateral SNHL.

## Materials and methods

### Patient recruitment and ethical considerations

This study was approved by the Ministry of Health and Prevention Research Ethics Committee, reference number MOHAP/DXB-REC/JJJ/No.71/2020, as per national regulations. Affected patients were identified by the otolaryngology team at Kuwait Hospital, Dubai, for clinical evaluation related to cochlear pre-implantation procedures. The inclusion criteria required patients to have a bilateral SNHL. Patients with other potential causes for bilateral SNHL, including infections, known syndromic conditions, or unilateral SNHL, were excluded from the analysis. A total of 11 families, each with one or more members diagnosed with bilateral SNHL, met the inclusion criteria and were recruited for this study during the period from March 2021 to January 2022. Informed written consent was obtained from all the participants in this study or from their parents or legal guardians if they were under the age of 18.

### Whole-exome sequencing and variant prioritization

In this study, we decided to run trio-whole-exome sequencing (WES) (proband, father, and mother) for all families except those with missing parental samples. For cases 8 and 10, we ran the WES on the proband and father and trio-WES (proband, father, and sister), respectively. The QIAcube instrument was used to extract genomic DNA from peripheral blood using the QIAamp DNA Blood Mini Kit (QIAGEN, Germany). WES was performed in the United Arab Emirates (UAE) University Genomics Laboratory, UAE. The DNA’s quality and quantity were determined using a NanoDrop One spectrophotometer (Thermo Fisher Scientific, USA) and a Qubit 3.0 fluorometer (Qubit dsDNA BR Assay; Thermo Fisher Scientific, USA), respectively. In brief, library preparation and target enrichment steps were carried out using TruSeq DNA Exome (Illumina, USA) in accordance with the manufacturer’s protocol. A Qubit 3.0 fluorometer (Qubit dsDNA HS Assay; Thermo Fisher Scientific, USA) and an Agilent 4200 TapeStation system (HS D1000 ScreenTape Assay; Agilent Technologies, USA) were used to determine the library’s concentration and fragment size, respectively. Using SP and S1 flow cells on the NovaSeq 6000 platform (Illumina, USA), the final normalized libraries were sequenced with paired-end reads (2 × 150 bp). A combination of in-house-developed pipelines and the Illumina DRAGEN Bio-IT Platform (Illumina, USA) was used for reads mapping, alignment, and variant calling. VarSeq 2.2.4 software (Golden Helix, USA) was used for variant annotation and filtration. The output data were further filtered against all disease-causing variants in ClinVar, the Human Genome Mutation Database (HGMD) (Stenson et al., 2003), and variants with a minor allele frequency (MAF) of less than 1% in the gnomAD database (Karczewski et al., 2020). Exonic and splice site variants in homozygous, hemizygous, compound heterozygous, and heterozygous states were investigated. Relevant inheritance patterns based on clinical information and family history provided by the referring physician were used to clinically correlate the identified variants. Filtered variants were interpreted using the American College of Medical Genetics and Genomics (ACMG) guidelines and patient phenotype (Richards et al., 2015). The identified pathogenic or likely pathogenic variants based on the ACMG recommendations were validated by Sanger sequencing during the study. Primer pairs, designed to flank the variant, were used with the Taq PCR Master Mix Kit (QIAGEN, Germany) to amplify the genomic DNA through PCR amplification (Supplementary Table S1). Following amplification, fluorescent automated sequencing was performed on the ABI 3130xl Genetic Analyzer (Applied Biosystems, USA) using the BigDye Terminator v3.1 Cycle Sequencing Kit (Applied Biosystems, USA).

### Computational analysis of variants

To predict the pathogenicity charge of missense variants, nine *in silico* tools were used: CADD (Combined Annotation-Dependent Depletion) (Rentzsch et al., 2019); REVEL (Rare Exome Variant Ensemble Learner) (Ioannidis et al., 2016); SIFT (Sorting Intolerant from Tolerant) (Sim et al., 2012); PolyPhen-2 (Polymorphism Phenotyping v2) (Adzhubei et al., 2010); PROVEAN (Protein Variation Effect Analyzer) (Choi et al., 2012); LRT (likelihood ratio test) (Chun and Fay, 2009); MutationTaster (Schwarz et al., 2014); MutationAssessor (Reva et al., 2011); and DANN (deleterious annotation of genetic variants using neural networks) (Quang et al., 2015). Additionally, the impact of missense variants on protein stability was assessed using I-Mutant (Capriotti et al., 2005). For this, the amino acid sequences of studied proteins were retrieved from UniProt, and substitutions at particular positions were introduced manually.

The evolutionary conservation of studied missense variants was evaluated using ConSurf (Ashkenazy et al., 2016). The amino acid sequences of each studied protein were obtained from UniProt and were used as input. Multiple sequence alignment (MSA) using ClustalW was also carried out to confirm the conservation of mutated residues at particular locations (Thompson et al., 1994). For this, human protein sequences were aligned and compared with chimpanzee, mouse, frog, rabbit, pig, bovine, elephant, chicken, and whale sequences.

The effect of point variants on protein structure and function was evaluated using the protein modeling approach. Modeller 10.1 was used to produce homology models of wild-type and mutant proteins where suitable templates were available (Webb and Sali, 2016). The protein sequences of studied human proteins were obtained from UniProt, and three-dimensional (3D) crystal structures retrieved from the Protein Data Bank (PDB) were used as templates to generate homology models of wild-type and mutant human protein structures. PyMOL was used to evaluate and visualize the generated models (DeLano, 2002).

## Results

### Clinical and genetic characteristics of the studied patients

In this study, WES was used to identify the genetic etiology of bilateral SNHL in families who presented to the otolaryngology clinic for cochlear implantation. A total of 11 families fulfilled this criterion and were further investigated. Variants contributing to the disease etiology were identified in the following genes that are known to be associated with HL.

#### MYO15A

**FIGURE 1:**
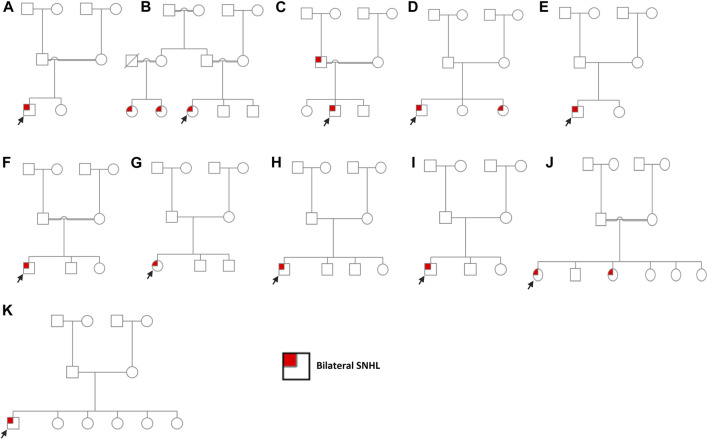
Pedigree charts of enrolled families. **(A)** Family 1. **(B)** Family 2. **(C)** Family 3. **(D)** Family 4. **(E)** Family 5. **(F)** Family 6. **(G)** Family 7. **(H)** Family 8. **(I)** Family 9. **(J)** Family 10. **(K)** Family 11. Males and females are represented by squares and circles, respectively. The probands are denoted by a small black arrow.

Case 1 is of a patient affected with bilateral SNHL. The patient was born prematurely (32 weeks of gestation) and was admitted to the neonatal intensive care unit (NICU) for 3 days after birth. His parents are first cousins with no prior family history of HL. The mother observed the hearing problem in the proband when he was 6 months old. Developmental milestones were found to be normal. He received right and left cochlear implants at the ages of 1 year and 3 years, respectively. WES was performed to determine the genetic reasoning of bilateral SNHL in the proband (Figure 1; Table 1). Results identified a novel homozygous nonsense variant, *MYO15A*:*p*.Tyr1962Ter* (Table 2). This variant has not been reported in any public database to the best of our knowledge. MutationTaster predicted this variant as pathogenic. It is classified as a likely pathogenic variant according to the recommendations of the ACMG.

**TABLE 1:** Clinical characteristics of the bilateral SNHL families and genotypes identified through WES.

Case/family ID	Proband gender	Age of diagnosis	Parents' consanguinity	Nationality/origin	Variant	Variant classification (ACMG guidelines)	Overall allele frequency (gnomAD)	Affected family members	Zygosity
1	Male	6 months	Yes	Egyptian	*MYO15A*:*p*.Tyr1962Ter*	Likely pathogenic (PVS1 and PM2)	NR	1	Homozygous
2	Female	1 year	Yes	Comorian	*SLC26A4*:*p*.Val239Asp	Pathogenic (PM1, PP3, PS3, and PS1)	2.03e-4	1	Homozygous
3	Male	6 months	Yes	Emirati	*SLC26A4*:*p*.Val239Asp	Pathogenic (PM1, PP3, PS3, and PS1)	2.03e-4	2	Homozygous
4	Male	0 months	No	Pakistani	*SLC26A4*:*p*.Trp83Ter*	Pathogenic (PM2, PVS1, and PM3)	NR	2	Compound heterozygous
					*SLC26A4*:*p*.Gln446Arg	Pathogenic (PS1, PS3, PM1, and PP3)	7.58e-5		
5	Male	1.5 years	No	Indian	*GJB2*:*p*.Trp24Ter*	Pathogenic (PVS1, PS3, and PM3_VS)	5.22e-4	1	Homozygous
6	Male	3 years	Yes	Egyptian	*GJB2*:*p*.Gly12Valfs*2	Pathogenic (PVS1, PS3, PM3, and PS4)	6.19e-3	1	Homozygous
7	Female	2 years	No	Egyptian	*CDH23*:*p*.Gly1025Asp	VUS (BS1 and PP3)	NR	1	Compound heterozygous
					*CDH23*:*p*.Arg2608His	VUS (BS1 and PP3)	NR		
8	Male	1.5 years	No	Afghani	*COL11A1*:*p*.Pro1077Thr	VUS (PM2 and BP4)	1.08e-4	1	Heterozygous
9	Male	1.5 years	No	Indian	*ADGRV1*:*p*.Leu2098Pro	VUS (PM2 and PP3)	NR	1	Heterozygous
10	Female	1.5 years	Yes	Pakistani	*NLRP3*:*p*.Ala879Gly	VUS (PM1, PM2, and BP4)	2.78e-5	2	Heterozygous
11	Male	2 years	No	Yemeni	*GDF6*:*p*.Ala435Val	VUS (PM1 and PP3)	1.77e-4	1	Heterozygous

SNHL, sensorineural hearing loss; WES, whole-exome sequencing; ACMG, American College of Medical Genetics and Genomics; VUS, variants of uncertain significance; NR, not reported.

**TABLE 2:** Computational analysis of identified variants in probands.

Gene	Variant	SIFT	PolyPhen-2	LRT	MutationTaster	MutationAssessor	PROVEAN	REVEL	CADD	DANN
		Score	Score	Score	Score	Score	Score	Score	Score	Score
*MYO15A*	*p*.Tyr1962Ter*	-	-	-	1 D	-	-	-	-	-
*SLC26A4*	*p*.Val239Asp	0.0 D	0.84 P	0 D	0.99 D	0.67 M	0.87 D	0.93 D	27 D	0.98 D
*SLC26A4*	*p*.Trp83Ter*	-	-	-	1 D	-	-	-	-	-
*SLC26A4*	*p*.Gln446Arg	0.24 T	1 D	0 D	0.99 D	0.83 M	0.61 D	0.91 D	27 D	0.99 D
*GJB2*	*p*.Trp24Ter*	-	-	-	1 D	-	-	-	-	-
*GJB2*	*p*.Gly12Valfs*2	-	-	-	1 D	-	-	-	-	-
*CDH23*	*p*.Gly1025Asp	0.04 D	0.87 D	0 D	1 D	0.7 M	0.81 D	0.98 D	27.5 D	0.99 D
*CDH23*	*p*.Arg2608His	0.33 B	0.91 D	0 D	1 D	0.2 N	0.47 D	0.75 D	29.3 D	0.99 D
*COL11A1*	*p*.Pro1077Thr	0.02 D	0.3 B	0 D	0.99 D	0.55 M	0.76 D	0.82 D	20.8 D	0.99 D
*ADGRV1*	*p*.Leu2098Pro	0.0 3D	1 D	0 D	1 D	1 D	0.78 D	0.80 D	27.1 D	0.99 D
*NLRP3*	*p*.Ala879Gly	0.01 D	0.53 P	0.03 D	1 D	0.60 M	0.57 D	0.5 M	12.3 D	0.83 D
*GDF6*	*p*.Ala435Val	0.0 D	0.90 D	0 D	0.99 D	0.31 L	0.66 D	0.75 D	32 D	0.99 D

SIFT, Sorting Intolerant from Tolerant. Lower scores indicate pathogenicity; PolyPhen-2, polymorphism phenotyping v2. Higher scores indicate pathogenicity; LRT, a likelihood ratio test based on two-sided *p*-value. LRT scores are computed using nonsynonymous-to-synonymous-rate ratio and alignment of amino acids of 31 species at the codon of interest. Scores range from 0 to 1, and lower scores indicate pathogenicity. MutationTaster uses different bioinformatics approaches to predict the pathogenicity of VUSs at the DNA and protein level. Higher scores indicate pathogenicity. MutationAssessor calculates the amino acid substitution impact on the protein using the conservation of the substituted residue in protein homologs. Higher scores indicate pathogenicity. PROVEAN, Protein Variation Effect Analyzer. Higher scores suggest pathogenicity. REVEL, Rare Exome Variant Ensemble Learner. Higher scores suggest pathogenicity. CADD, Combined Annotation-Dependent Depletion. Scores range from 1 to 99. Higher scores signify pathogenicity; e.g., a score of 30 indicates a 0.1% top variant. DANN, deleterious annotation of genetic variants using neural networks. Higher scores indicate pathogenicity. B, benign; D, deleterious; H, high; L, low; T, tolerated; M, medium.

#### SLC26A4

*SLC26A4:p*.Val239Asp was identified in probands of two families (case 2 and case 3) in a homozygous state. Additionally, it was detected in the affected father with bilateral SNHL in case 3 (Figure 1; Supplementary Figure S1). The proband in case 2 was diagnosed with bilateral SNHL at the age of 1 year. Her perinatal and postnatal milestones were normal except for delayed speech. The proband in case 3 was diagnosed with bilateral SNHL at the age of 6 months. He was born prematurely (35 weeks of gestation) and spent 15 days in the NICU after birth. Both probands' parents were first cousins with a history of HL (Figure 1; Table 1). Computational tools consistently predicted *SLC26A4*:*p*.Val239Asp as pathogenic (Table 2). Val239 is a highly conserved residue. According to the ClinVar classification based on the ACMG criteria, this variant is classified as pathogenic, and therefore, it is considered a pathogenic variant.

In case 4, on the other hand, the patient had two variants in a compound heterozygous state in the *SLC26A4* gene. The stop-gained variant *p*.Trp83Ter* and the missense variant *p*.Gln446Arg were of maternal and paternal origin, respectively (Supplementary Figure S1). He had bilateral HL since birth and was put on hearing aids at the age of 4 years. His developmental milestones were found to be normal. He received a left cochlear implant at the age of 6 years. The proband’s parents are non-consanguineous (Figure 1; Table 1). The presence of both *SLC26A4* variants has also been confirmed in the patient’s similarly affected sister through WES. MutationTaster predicted these variants to be pathogenic (Table 2). ClinVar classifies both variants as pathogenic based on the ACMG recommendations. Thus, these two variants are considered pathogenic.

#### GJB2

*GJB2*:*p*.Trp24Ter* was found in case 5 in a homozygous state. The patient had a positive history of bilateral SNHL (diagnosed at the age of 1.5 years) with no prior history of prenatal infection, meningitis, hyperbilirubinemia, or NICU admission. His developmental milestones were observed to be normal. The family had no history of HL (Figure 1; Table 1). MutationTaster predicted this variant as pathogenic (Table 2). Based on the ACMG criteria, as classified by ClinVar, it is pathogenic. Thus, the variant is considered pathogenic. *GJB2*:*p*.Gly12Valfs*2 was found in case 6 in a homozygous state. The proband was diagnosed with bilateral SNHL at the age of 3 years with negative perinatal and postnatal milestones. His parents were first cousins without a family history of HL (Figure 1; Table 1). Computational tools predicted *GJB2*:*p*.Gly12Valfs*2 as pathogenic (Table 2). ClinVar classifies this variant as pathogenic based on the ACMG recommendations, and therefore, it is pathogenic.

#### CDH23

*CDH23*:*p*.Gly1025Asp and *CDH23*:*p*.Arg2608His variants were observed in case 7 in a compound heterozygous state from trio-WES analysis (proband, father, and mother). The proband was diagnosed with bilateral SNHL at the age of 2 years. She was found to be walking slowly; this was probably due to flat feet. She also had a balance problem, possibly indicating ataxia. Her parents are non-consanguineous, and there was no family history of HL (Figure 1; Table 1). *In silico* tools consistently predicted these variants as pathogenic (Table 2). Based on the ClinVar classification and ACMG criteria, both variants are of uncertain significance (VUS). In this case, the clinical information of the patient and computational results of *CDH23*:*p*.Gly1025Asp and *CDH23*:*p*.Arg2608His confirm the pathogenicity of these variants.

#### VUSs identified in additional clinically diagnosed SNHL patients

*COL11A1*:*p*.Pro1077Thr, *ADGRV1*:*p*.Leu2098Pro, *NLRP3*:*p*.Ala879Gly, and *GDF6*:*p*.Ala435Val variants were identified in a heterozygous state in cases 8–11, respectively. *In silico* tools consistently predicted them as deleterious, except for *NLRP3*:*p*.Ala879Gly (Table 2), likely due to the low scores predicted by CADD, PolyPhen-2, and LRT tools. Based on ACMG criteria, these four variants are VUSs, and thus, their clinical significance is not known. Cases 8, 9, and 10 were clinically diagnosed with bilateral SNHL at the age of 1.5 years, and their developmental milestones were observed as normal. In cases 8 and 9, the probands' parents are non-consanguineous, while in case 10, they are consanguineous. (Figure 1; Table 1). Case 8, 9, and 10 received right cochlear implants at the age of 4, 10, and 7 years, respectively. In case 10, one sister has bilateral SNHL, while the other siblings are healthy. Similar to the proband, this affected sister had the variant *NLRP3*:*p*.Ala879Gly detected, present in a heterozygous state. The proband in case 11 was diagnosed with bilateral SNHL when he was 2 years old. His developmental milestones were found to be delayed. He was also found to have autosomal recessive spinocerebellar ataxia with peripheral neuropathy. His other siblings (five sisters) are all healthy, and his parents are non-consanguineous (Figure 1; Table 1). *ADGRV1:p*.Leu2098Pro (case 9) and *GDF6:p*.Ala435Val (case 11) were found in probands in a heterozygous state, inherited from their asymptomatic mothers. Additionally, the inheritance of *COL11A1*:*p*.Pro1077Thr (case 8) and *NLRP3*:*p*.Ala879Gly (case 10) is unknown because the mothers’ samples were not available for testing.

### *In silico* pathogenicity analysis of the identified variants

In this study, 12 variants were observed in eight genes. The effect of variants on putative post-translational modification (PTM) sites was also investigated. Nevertheless, no variants were found at sites identified as post-translationally modified. Three nonsense variants were found in *MYO15A*, S*LC26A4*, and *GJB2* genes and were predicted to be pathogenic (Table 2). *MYO15A*:*p*.Tyr1962Ter* is a novel homozygous variant that leads to a premature stop codon at amino acid position 1962 for the MYO15A protein, whereas the *GJB2*:*p*.Trp24Ter* and *SLC26A4*:*p*.Trp83Ter* variants result in premature stop codons at amino acid positions 24 and 83 for the GJB2 and SLC26A4 proteins, respectively.

This study also identified eight missense variants. Computational tools consistently predicted these missense variants as pathogenic (Table 2). *ADGRV1*:*p*.Leu2098Pro has not been reported in any public database. Another variant, where leucine was substituted with phenylalanine at this position, has been reported as VUS in ClinVar (VCV001054831.3). Therefore, the clinical significance of *ADGRV1*:*p*.Leu2098Pro is not known.

### Evolutionary conservation analysis

**FIGURE 2:**
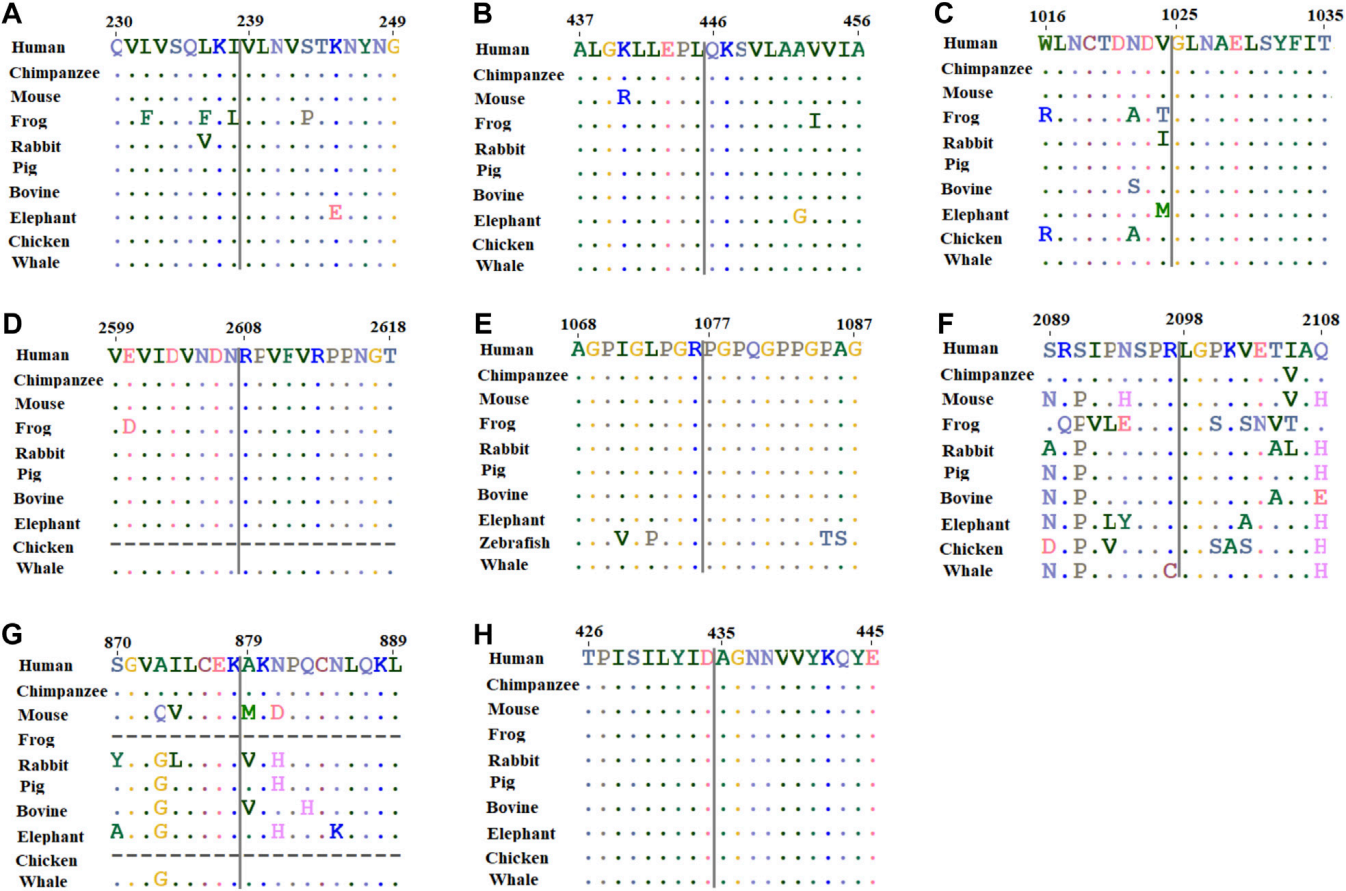
Multiple sequence alignment of amino acids located around the missense variant obtained from human, chimpanzee, mouse, frog, rabbit, pig, bovine, elephant, chicken, and whale sequences where available. **(A)** *SLC26A4*:*p*.Val239; **(B)** *SLC26A4*:*p*.Gln446; **(C)** *CDH23*:*p*.Gly1025; **(D)** *CDH23*:*p*.Arg2608; **(E)** *COL11A1*:*p*.Pro1077; **(F)** *ADGRV1*:*p*.Leu 2098; **(G)** *NLRP3*:*p*.Ala879; and **(H)** *GDF6*:*p*.Ala435.

In order to assess the conservation of mutated residues in the studied protein, we carried out ConSurf analysis. The location of the variants in the studied protein in the wild-type sequences is highlighted in the blue-outlined box (Supplementary Figure S2). To further confirm the conservation of substituted residues predicted by ConSurf, MSA was performed using ClustalW. For MSA, amino acid sequences of humans and different organisms were obtained from UniProt and aligned (Supplementary Table S2). The results revealed that *SLC26A4*:*p*.Val239, *SLC26A4*:*p*.Gln446*, CDH23*:*p*.Gly1025, *CDH23*:*p*.Arg2608, *COLL11A1*:*p*.Pro1077, *ADGRV1*:*p*.Leu 2098, and *GDF6*:*p*.Ala435 amino acids are conserved, but *NLRP3*:*p*.Ala879 is not (Figure 2). As a result, any substitution at these positions is likely to have a deleterious impact on protein structure and function.

### Structural stability analysis and impact of variants on protein structures and functions

The effect of missense variants on protein stability was assessed using I-mutant. Overall, all these missense variants decreased protein stability when compared to wild-type variants, with different reliability index scores (Table 3). Among the eight missense variants, *ADGRV1*:*p*.Leu2098Pro, *CDH23*:*p*.Arg2608His, and *SLC26A4*:*p*.Val239Asp had the highest instability in their respective proteins, with a Gibbs free energy change value (ΔΔG) of −1.62, −1.57, and −1.38, respectively (Table 3).

**TABLE 3:** Protein stability analysis predicted by I-mutant suite.

Variant	Stability	RI (0–10)	ΔΔG (Kcal/mol)
*SLC26A4*:*p*.Val239Asp	Decrease	7	−1.38
*SLC26A4*:*p*.Gln446Arg	Decrease	1	−0.59
*CDH23*:*p*.Gly1025Asp	Decrease	9	−1.31
*CDH23*:*p*.Arg2608His	Decrease	9	−1.57
*COL11A1*:*p*.Pro1077Thr	Decrease	3	−0.63
*ADGRV1*:*p*.Leu2098Pro	Decrease	5	−1.62
*NLRP3*:*p*.Ala879Gly	Decrease	9	−1.22
*GDF6*:*p*.Ala435Val	Decrease	4	−0.18

RI, reliability index; ΔΔG, change in Gibbs free energy. ΔΔG <0 indicates a decrease in stability, while ΔΔG >0 indicates an increase in stability.

**FIGURE 3:**
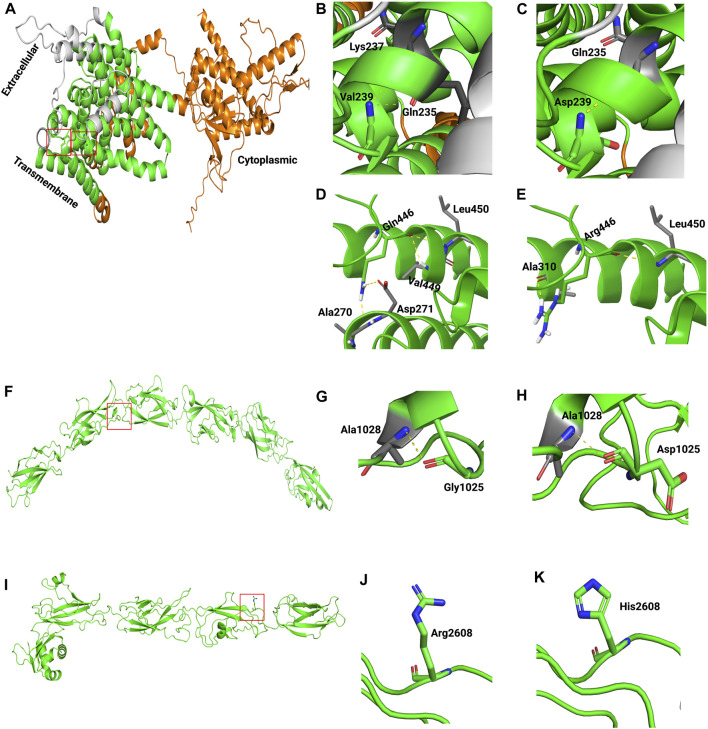
Generated homology models of pathogenic missense variants in SLC26A4 and CDH23. The proteins are depicted in cartoon form, while the amino acids are represented as sticks. In the following images, the boxed area in A, F, and I are magnified. **(A)** Modeled structure of SLC26A4. **(B)** Wild-type SLC26A4:*p*.Val239. **(C)** Mutant SLC26A4:*p*.Asp239. **(D)** Wild-type SLC26A4:*p*.Gln446. **(E)** Mutant SLC26A4:*p*.Arg446. **(F)** Modeled structure of CDH23. **(G)** Wild-type CDH23:*p*.Gly1025. **(H)** Mutant CDH23:*p*.Asp1025. **(I)** Modeled structure of CDH23. **(J)** Wild-type CDH23:*p*.Arg2608. **(K)** Mutant CDH23:*p*.His2608.

Last, to assess the effect of missense variants on protein structure and function, 3D structures of wild-type and mutants of human SLC26A4, CDH23, ADGRV1, NLRP3, and GDF6 were modeled (Figure 3; Supplementary Figure S3). The templates used for modeling these proteins are presented in Supplementary Table S3. Because no suitable template for COL11A1 was available, its 3D structure was not modeled. Structural analysis indicated that wild-type SLC26A4:*p*.Val239 formed two hydrogen bonds with Gln235 and Lys237. However, the introduction of charged residue Asp239 only resulted in a hydrogen bond with Gln235 (Figures 3B, C). Wild-type SLC26A4:*p*.Gln446 formed four hydrogen bonds, but three of these hydrogen bonds were lost when arginine residue was introduced at this position (Figures 3D, E). It is perceivable that this change could likely make the protein unstable. *CDH23*:*p*.Gly1025Asp and *CDH23*:*p*.Arg2608His were found in cadherin 10 and cadherin 24 domains of the cadherin-23 protein, respectively. The changes were physiochemically significant. Gly1025Asp and Arg2608His were found in conserved Ca2^+^-binding motifs DXD and DXNDN in the loop regions of CDH23, respectively. Previous studies have shown that variants in Ca2^+^-binding motifs have impacted the unfolding strength and flexibility of the linker region as well as altered the Ca2^+^ affinity, resulting in decreased mechanical strength at the physiological Ca2^+^ concentrations of cochlear endolymph (Courjean et al., 2008; Sotomayor et al., 2010). Variants in ADGRV1, NLRP3, and GDF6 were found to be physiochemically significant, but they likely formed local changes (Supplementary Figure S3).

## Discussion

In this study, we identified genetic variants associated with bilateral SNHL using the WES approach in 11 families residing in the UAE. Approximately 45.5% of the patients came from consanguineous families. We established the molecular diagnosis in six probands, with six different variants in three HL-associated genes. We also found one novel nonsense variant, *MYO15A*:*p*.Tyr1962Ter*, in a homozygous state in one proband, which has not been reported in any public database so far. A recent study reported another novel variant in the *MYO15A* gene in an Arab population (Asaad et al., 2023), reflecting the importance of conducting further studies to unveil additional genetic variants in *MYO15A* causing SNHL, particularly in Arab populations. The diagnostic rate of this study (54.5%) was comparable to a recent study (60.4%) (Uehara et al., 2022) and relatively higher than that of earlier studies (39.3% and 30.0%) (Mori et al., 2016; Sloan-Heggen et al., 2016). This high diagnostic rate is most likely caused by the cohort’s strong family history of HL. In fact, 36.4% of our cohort had a history of HL in their families. Of note, a study has demonstrated that sporadic cases have a lower diagnostic rate (19.0%) compared to autosomal dominant (35.0%) and autosomal recessive (35.0%) cases (Moteki et al., 2016). From a diagnostic standpoint, these clinical attributes could be helpful in prioritizing candidate genes for genetic testing in order to improve diagnostic yield.

A previous study demonstrated that causal genes vary between early-onset or congenital HL and late-onset HL (Mori et al., 2016). We also found multiple responsible genes that have been linked to early-onset HL. Moreover, the HL from these genes is mostly progressive. The rate of disease progression and its underlying causes play crucial roles in determining the appropriate course of management for any condition. This is where genetic testing may provide valuable information to guide these management decisions. When the root cause of HL is localized within the cochlea, cochlear implantation emerges as a viable intervention. In such cases, genetic testing not only aids in identifying the etiology but also provides insights into the potential success of cochlear implantation (Usami et al., 2020). Genetic variants of the *CDH23* gene have been linked to Usher syndrome type 1D (USH1D) and non-syndromic HL. The phenotype presentation of HL associated with *CDH23* ranges from congenital to adult-onset HL (Miyagawa et al., 2012). We also observed a case with *CDH23* variants who performed well after receiving a cochlear implant.

Most *MYO15A* variants have been linked with a congenital severe-to-profound HL phenotype. Studies have found the prevalence of *MYO15A* variants among autosomal recessive non-syndromic HL patients from 3% to 10% in different populations (Fattahi et al., 2012; Richard et al., 2019). Here, we found a case with a nonsense variant in *MYO15A* who demonstrated profound bilateral SNHL.

*SLC26A4* variants have been identified as the second most common cause of deafness in various populations, particularly in Asian countries (Park et al., 2003; Yan et al., 2013; Yan et al., 2016). Variants in *SLC26A4* have been linked to syndromic deafness characterized by congenital SNHL and goiter (Pendred syndrome) (Dossena et al., 2011; Najmabadi and Kahrizi, 2014). We found *SLC26A4*:*p*.Val239Asp in a homozygous state in two families in one patient each and *SLC26A4*:*p*.Trp83Ter* and *SLC26A4*:*p*.Gln446Arg variants together in a compound heterozygous state in two patients of one family. The frequency of *SLC26A4* variants in this study was observed to be 27.3%.

The genetic variants of *GJB2* are the most common cause of congenital HL, and its variant spectrums differ between ethnic groups (Ohtsuka et al., 2003; Downie et al., 2020). To provide proper genetic counseling, it is critical to investigate the carrier frequency and variant spectrum of each genetic background. In this study, *GJB2*:*p*.Trp24Ter* and *GJB2*:*p*.Gly12Valfs*2 were observed in one family each. The frequency of *GJB2* variants was found to be approximately 18.2%.

This study also reports six VUSs (50.0%) based on the ACMG criteria. Our extensive *in silico* pathogenicity prediction pipeline predicted them as likely pathogenic. We were not able to determine the inheritance of two variants, *COL11A1*:*p*.Pro1077Thr (case 8) and *NLRP3*:*p*.Ala879Gly (case 10), as the mothers’ samples were not available for confirmatory testing, and the variants were not detected in either father. *ADGRV1:p*.Leu2098Pro (case 9) and *GDF6:p*.Ala435Val (case 11) were found in probands in a heterozygous state through trio-WES analysis, which they inherited from their asymptomatic mothers. In case 11, it is plausible that the proband might have a second variant in the non-coding region of *GDF6*, which could further elucidate the patient’s genotype–phenotype relationship compared to the mother. A recent study reported a loss-of-function variant in the non-coding region of *GDF6* in two families affected with non-syndromic HL (Bademci et al., 2020). The study provided supportive molecular evidence signifying the role of *GDF6* in early cochlear development. In our study, further comprehensive family segregation is needed to confirm their pathogenicity and involvement in bilateral SNHL. Alternatively, considering whole-genome sequencing (WGS) would enable investigating the regulatory elements of *GDF6*.

Understanding the genetic basis of HL provides an insight into the clinical diagnosis of hearing impairment cases utilizing NGS. Our findings demonstrate that genetic diagnosis is achievable, especially within families experiencing inherited HL in a population characterized by a high prevalence of consanguineous marriages within Arab communities. These diagnoses can predict syndromic effects, aiding in treatment decisions and providing insights for prognosis counseling. They can also give parents the chance to receive a pre-conception diagnosis for upcoming pregnancies. Our findings contribute to the understanding of the heterogeneous genetic profile of HL, aligning with many other studies (Abu Rayyan et al., 2020; Molina-Ramírez et al., 2021) that demonstrated the reproducibility of high rates of genetic diagnoses within affected families across various populations.

## Figures and Tables

**Table:** 

**HL**	Hearing loss
**SNHL**	Sensorineural hearing loss
**WES**	Whole-exome sequencing
**UAE**	United Arab Emirates
***MYO15A***	Myosin XVA gene
***SLC26A4***	Solute carrier family 26 member 4 gene
***GJB2***	Gap junction protein beta 2 gene
**VUS**	Variant of uncertain significance
***CDH23***	Cadherin-related 23 gene
***COL11A1***	Collagen type XI alpha 1 chain gene
***ADGRV1***	Adhesion G protein-coupled receptor V1 gene
***NLRP3***	NLR family pyrin domain containing 3 gene
***GDF6***	Growth differentiation factor 6 gene
**NGS**	Next-generation sequencing
**OMIM**	Online Mendelian Inheritance in Man
**ClinVar**	Clinical Variation Database
**HGMD**	Human Genome Mutation Database
**MAF**	Minor allele frequency
**gnomAD**	Genome Aggregation Database
**ACMG**	American College of Medical Genetics and Genomics
**PCR**	Polymerase chain reaction
**CADD**	Combined Annotation-Dependent Depletion
**REVEL**	Rare Exome Variant Ensemble Learner
**SIFT**	Sorting Intolerant from Tolerant
**PolyPhen-2**	Polymorphism phenotyping v2
**PROVEAN**	Protein Variation Effect Analyzer
**LRT**	Likelihood ratio test
**DANN**	Deleterious annotation of genetic variants using neural networks
**MSA**	Multiple sequence alignment
**PDB**	Protein Data Bank
**NICU**	Neonatal intensive care unit
**PTM**	Post-translational modification
**USH1D**	Usher syndrome type 1D
**WGS**	Whole-genome sequencing

## Data Availability

The datasets presented in this study can be found in online repositories. The names of the repository/repositories and accession number(s) can be found in the article/Supplementary Material.
