# Supplementary material for: Spectrum of genetic variants in bilateral sensorineural hearing loss
Source: Front Genet. 2024 Feb 12;15:1314535. doi: 10.3389/fgene.2024.1314535 (PMC10894970; doi:10.3389/fgene.2024.1314535)
Supplement: Supplementary file 1 [file DataSheet1.docx]

**Supplementary Material**


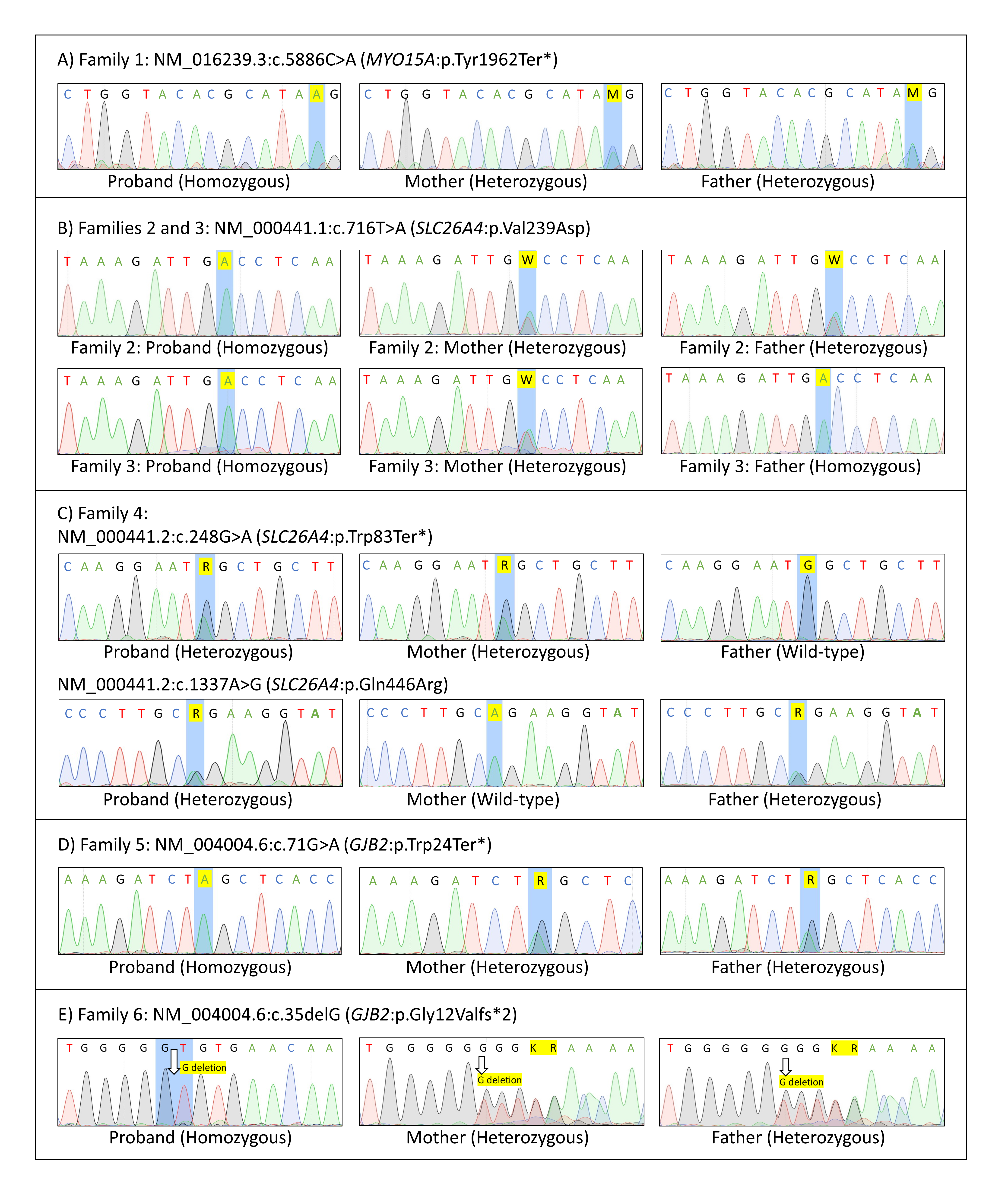


**Supplementary Figure 1.** Sanger sequencing electropherograms of identified pathogenic/likely pathogenic variants in study families. A) Family 1; B) Families 2 and 3; C) Family 4; D) Family 5; E) Family 6.


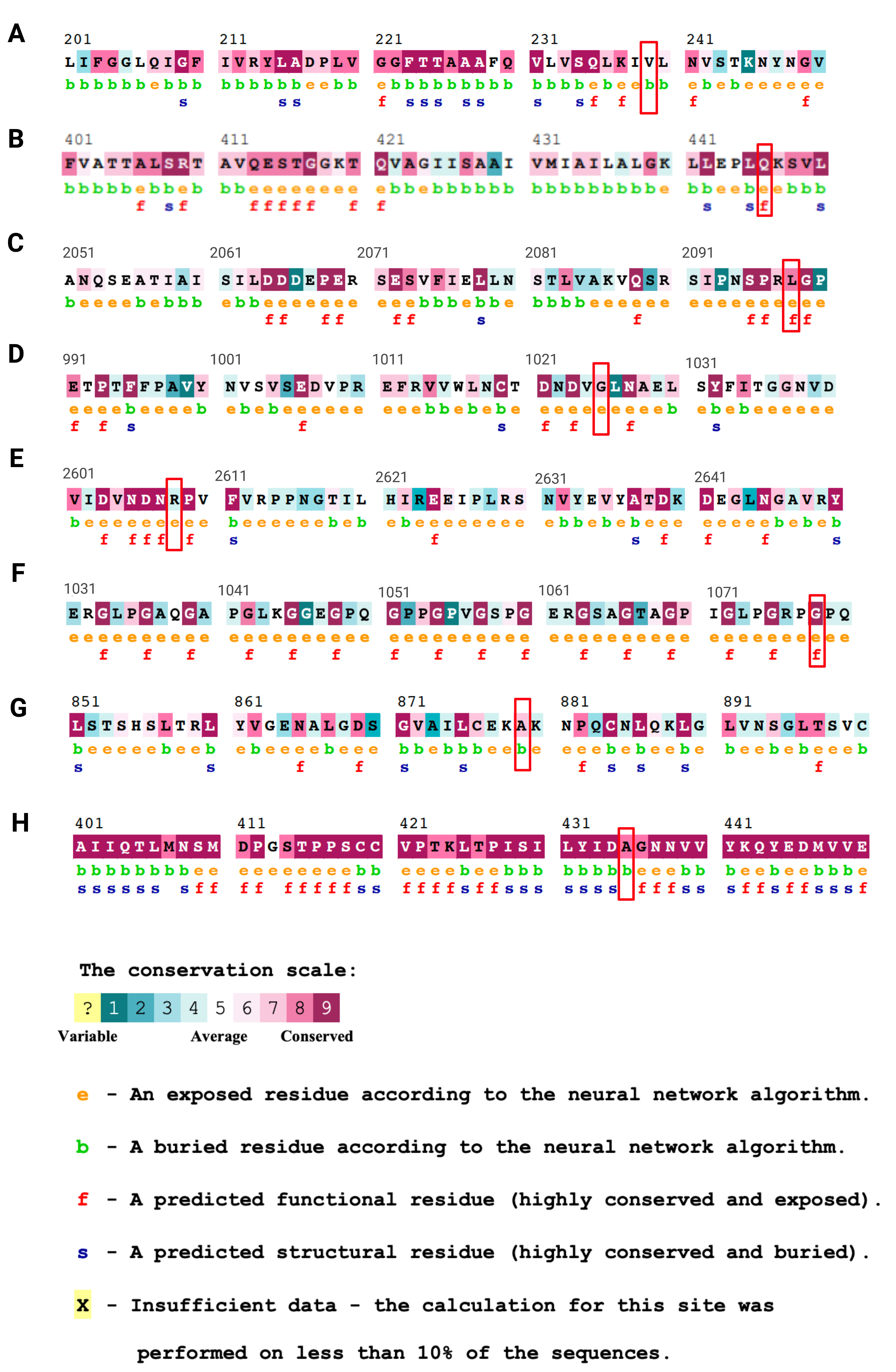


**Supplementary Figure 2.** Conservation of amino acid predicted by ConSurf. For the sake of space only 50 residues around studied wild type residue are shown. Square box shown in red color represents the location of mutation. A) SLC26A4:p.Val239; B) SLC26A4:p.Gln446; C) ADGRV1:p.Leu2098; D) CDH23:p.Gly1025; E) CDH23:p.Arg2608; F) COL11A1:p.Pro1077; G) NLRP3:p.Ala879; H) GDF6:p.Ala435.


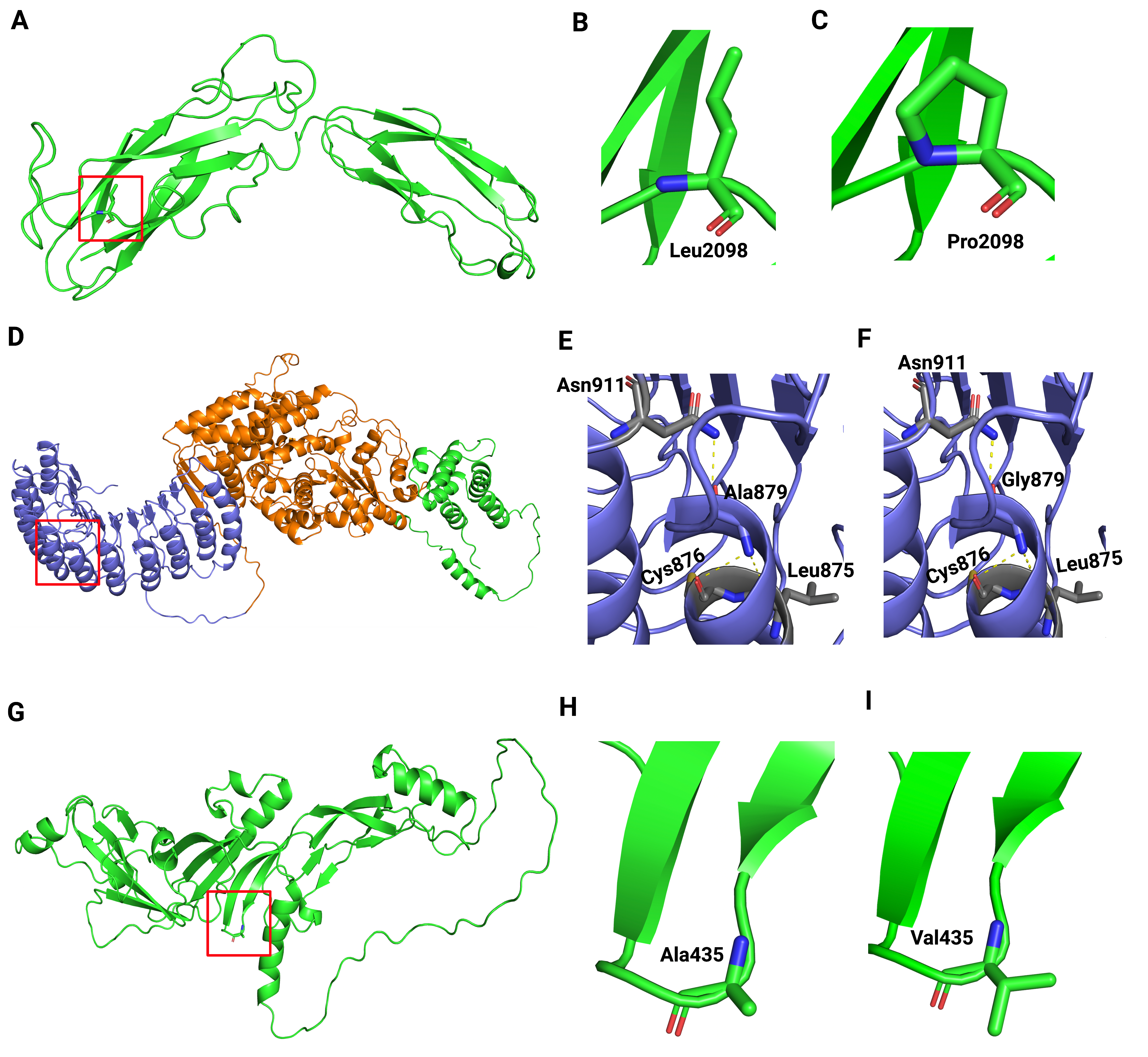


**Supplementary Figure 3.** The generated homology models of studied missense variants. The proteins are depicted in cartoon form, while the amino acids are represented as sticks. In the following images, the boxed area in A, D, G, and J are magnified. A) Modeled structure of ADGRV1; B) Wild type ADGRV1:p.Leu2098; C) Mutant ADGRV1:p.Pro2098; D) Modeled structure of NLRP3; E) Wild type NLRP3:p.Ala879; F) Mutant NLRP3:p.Gly879; G) Modeled structure of GDF6; H) Wild type GDF6:p.Ala435; I) Mutant GDF6:p.Val435.

**Supplementary Table 1.** List of primer pairs used in Sanger sequencing confirmation of pathogenic/likely pathogenic variants.

| Variant | Gene | Primer | Primer sequence (5'-3') | Length | TM | Amplicon size (bp) |
| --- | --- | --- | --- | --- | --- | --- |
| *MYO15A*:p.Tyr1962Ter* | *MYO15A* | *MYO15A4* Ex25 FWD | CCCAAAAGTGAGATGTGGGC | 20 | 60 | 293 |
|  | *MYO15A* | *MYO15A4* Ex25 REV | TGCCTGAGGCCAGTAGTCTC | 20 | 62 |  |
| *SLC26A4*:p.Val239Asp | *SLC26A4* | *SLC26A4* Ex6 FWD | GCAGATCCTTTGGTTGGTGG | 20 | 62 | 220 |
|  | *SLC26A4* | *SLC26A4* Ex6 REV | TGGCCCAGACTCAGAGAATG | 20 | 62 |  |
| *SLC26A4*:p.Trp83Ter* | *SLC26A4* | *SLC26A4* Ex3 FWD | GGTTGTGACTGAGATTGGATTG | 22 | 59 | 269 |
|  | *SLC26A4* | *SLC26A4* Ex3 REV | TGTCACAGACCTATGGTAGCTG | 22 | 57 |  |
| *SLC26A4*:p.Gln446Arg | *SLC26A4* | *SLC26A4* Ex11 FWD | CAGTGAGCTGGAAGACACAAGG | 22 | 62 | 269 |
|  | *SLC26A4* | *SLC26A4* Ex11 REV | CAGAGAAGGCTGTGTTATTCAG | 22 | 57 |  |
| Both: *GJB2*:p.Trp24Ter*; and *GJB2*:p.Gly12Valfs*2 | *GJB2* | *GJB2* EX2 FWD | GTCCTAGCTAGTGATTCCTGTG | 23 | 60 | 274 |
|  | *GJB2* | *GJB2* EX2 REV | GAAGTAGTGATCGTAGCACACG | 22 | 62 |  |

**Supplementary Table 2.** UniProt IDs of protein sequences used for performing multiple sequence alignment.

| Protein | UniProt ID |
| --- | --- |
| SLC26A4 | O43511 |
| CDH23 | Q9H251 |
| ADGRV1 | Q8WXG9 |
| NLRP3 | Q96P20 |
| GDF6 | Q6KF10 |
| COL11A1 | P12107 |

**Supplementary Table 3.** List of templates used for modeling studied protein.

| Protein | Template (PDB ID) | Organism | Sequence Identity (%) |
| --- | --- | --- | --- |
| SLC26A4 | 7WL9 | Mouse | 87.5 |
| CDH23 | 7SZ8 | Human | 36.59 |
| CDH23 | 5VT8 | Mouse | 86.6 |
| ADGRV1 | 3US9 | Dog | 30.2 |
| GDF6 | 5HLY | Homo sapiens | 28.4 |
